# Applications of targeted proteomics in systems biology and translational medicine

**DOI:** 10.1002/pmic.201500004

**Published:** 2015-07-16

**Authors:** H. Alexander Ebhardt, Alex Root, Chris Sander, Ruedi Aebersold

**Affiliations:** ^1^ Department of BiologyInstitute of Molecular Systems BiologyEidgenossische Technische Hochschule (ETH) ZurichZurichSwitzerland; ^2^ Computational Biology CenterMemorial Sloan‐Kettering Cancer CenterNew YorkNYUSA; ^3^ Department of PhysiologyBiophysics and Systems BiologyWeill Cornell Medical CollegeNew YorkNYUSA; ^4^ Faculty of ScienceUniversity of ZurichZurichSwitzerland

**Keywords:** Clinical proteomics, Multiple reaction monitoring, Selected reaction monitoring, Systems biology, Targeted proteomics

## Abstract

Biological systems are composed of numerous components of which proteins are of particularly high functional significance. Network models are useful abstractions for studying these components in context. Network representations display molecules as nodes and their interactions as edges. Because they are difficult to directly measure, functional edges are frequently inferred from suitably structured datasets consisting of the accurate and consistent quantification of network nodes under a multitude of perturbed conditions. For the precise quantification of a finite list of proteins across a wide range of samples, targeted proteomics exemplified by selected/multiple reaction monitoring (SRM, MRM) mass spectrometry has proven useful and has been applied to a variety of questions in systems biology and clinical studies. Here, we survey the literature of studies using SRM‐MS in systems biology and clinical proteomics. Systems biology studies frequently examine fundamental questions in network biology, whereas clinical studies frequently focus on biomarker discovery and validation in a variety of diseases including cardiovascular disease and cancer. Targeted proteomics promises to advance our understanding of biological networks and the phenotypic significance of specific network states and to advance biomarkers into clinical use.

**Abbreviations**
AIMS: accurate inclusion mass scanning
CPTAC: clinical proteomics tumor analysis consortium
ESI: electrospray ionization
HPLC: high pressure liquid chromatography
MRM: multiple reaction monitoring
MS: mass spectrometry
MS/MS: tandem mass spectrometry
PTM: post‐translational modification
RPPA: reverse‐phase protein array
RT: retention time
SID: stable isotope dilution
SISCAPA: stable isotope standards and capture by anti‐peptide antibodies
SRM: selected reaction monitoring
QQQ: triple quadrupole

## 1 Introduction

### 1.1 Why networks?

**Figure 1:**
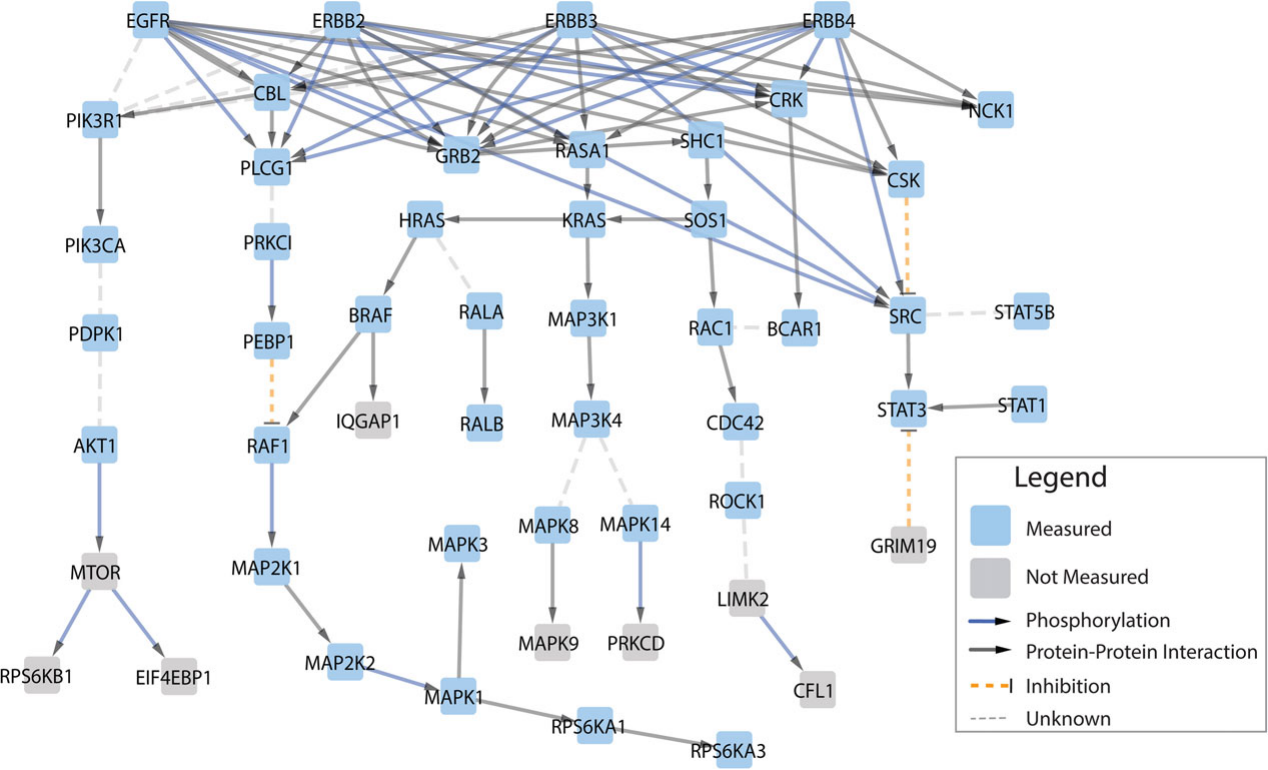
Network biology paradigm and complexities of proteomes. **(A)** Network biology paradigm. Protein–protein interactions can be modeled as networks involving a variety of interaction types. **(B)** A few complexities of the proteome. Studying proteins is complicated due to several factors: (i) a typical cell contains in excess of 20 000 different proteins, isoforms, and post‐translational modifications (PTMs); (ii) the range of absolute abundances spans more than seven orders of magnitude; (iii) each cell, tissue, and organism has a different complement of proteins; (iv) proteins vary in space and (v) in time; (vi) proteins are involved in numerous interactions subject to context‐dependent “rewiring”.

Many observations related to signaling cascades and other biological processes cannot be explained with a simple linear network model. This has led to the realization that the components of biological systems are not simply connected in a linear fashion but through a web of interactions, feedback loops, and crosstalk at multiple spatial and temporal scales and prompted a shift towards considering these processes as dynamic network. Models of such networks aim at increasing our understanding how changes in network state in specific contexts throughout normal development, disease, and in response to perturbations 1, 2, 3 generate or modulate phenotypes. A molecular network is an abstract construct in which nodes represent molecules and the edges signify a variety of physical or functional types of interactions. Physical interactions include interactions between proteins, enzyme–substrate relationships, transcription factors and their target genes, and protein–RNA interactions. Functional interactions include transient enzyme–substrate interactions, genetic interactions7&N and other functional dependencies of presently unknown mechanism. Because functional interactions are difficult to directly measure they are frequently inferred by statistical correlation from suitably structured, large datasets 4, 5, 6. Networks also provide a unifying conceptual and mathematical framework for systems biology, ecology, and neuroscience 7. An example of a biological network from the recent literature 8 is shown in Fig. 1. Alongside conceptual advances, immense technological progress now supports the identification and quantification of nucleic acids, proteins, lipids, carbohydrates, metabolites, and small molecules at sufficient coverage, depth, and throughput for the study of biological networks in diverse contexts, such as cell lines, body fluids, or tissues from various organisms and across developmental stages or among phenotypic states 9.

### 1.2 Why proteins?

**Figure 2:**
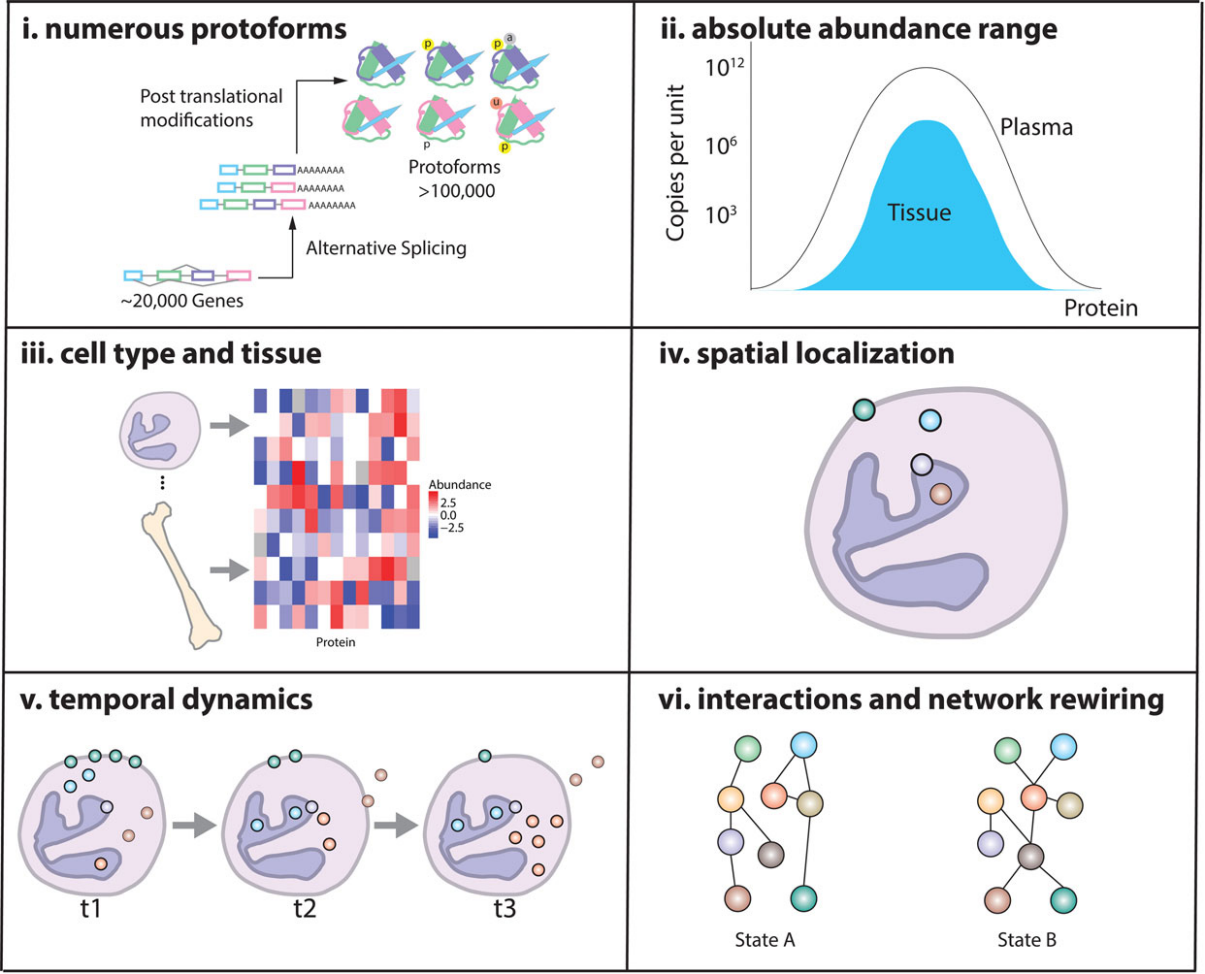
Proteins vary greatly within the cell. There are numerous protoforms to consider which arise from alternative splicing of pre‐mRNA and post‐tranlational modifications (i). The absolute abundance range of proteins is different in tissue than plasma (ii). Within each cell type, different proteomes are expressed (iii). The spatial localization of proteins also effects the proteins activity (iv). As a function of time and/or stimulus, protein levels and/or spacial distribution might differ (v). The activity of proteins is effected by protein‐protein interactions and rewiring of protein networks (vi). All points raised above effect methods to extract the proteome, or parts thereof.

Proteins are key components of many types of molecular networks, perform most biochemical functions of the cell and are the targets of most current drugs 10. Although proteins can be reliably identified by the well known discovery proteomics methods at high throughput 11 and their 3D structures can be determined experimentally or computationally 12, there remain numerous unsolved problems relating to their structure and function in the context of network biology. Measuring proteins poses technical challenges, particularly in higher eukaryotes which are made‐up of trillions of cells that are categorized, somewhat arbitrarily, into more than 400 different cell types 13. The two foremost challenges are the sheer number of proteins present in a cell and their vast dynamic range of expression which spans four to five orders of magnitude in prokaryotes, —six to seven orders of magnitude in eukaryotic cells/tissues and 12 orders of magnitude in body fluids 14, 15, 16. Moreover, proteins are subject to more than 200 types of PTMs, which further magnify the number of distinct protein entities in a sample and sample handling chemistries 17, 18. Figure 2 illustrates these challenges. In all, from a single protein coding genomic loci a myriad of protoforms can arise, especially in higher eukaryotes, which can be identified and quantified using high mass accuracy MS.

Protein networks pose a number of data analysis challenges that are rooted in the fact that proteins cannot be represented as simple molecular entities. Rather, they operate in a large number of biological contexts, spatial, and temporal scales and functional states exemplified by protein‐specific properties such as reaction mechanisms, substrate/ motif binding and complex formation. In addition, properties of protein networks such as information processing, noise, adaptability, robustness, and even seemingly paradoxical arrangement of components and functions, such as enzyme promiscuity 19, 20, 21, 22, 23, 24, 25, 26. Moreover, a protein can exist with different variant sequences due to splicing or mutations, and be subject to different PTMs at different sites, resulting in a vast number of theoretical combination of PTMs, known as “mod‐forms”, see for example the histone‐code 18. Even for a protein as well studied as Akt, a recent study found PTMs affecting a new layer of activation mechanism in cell cycle progression 27. Therefore, while there are compelling reasons to study protein networks of the cell, their analysis challenges current algorithmic and technical capabilities.

### 1.3 Protein identification and quantification

There are two main ways to detect and quantify proteins: affinity reagent based methods, exemplified by ELISA, Western blotting or immuno histochemistry staining, and MS based peptide identification and quantification, which is mainly used for research and discovery proteomics. However, the dynamic range and number of proteins quantifiable using affinity‐reagent based assays is limited 28. Quantitatively describing protein networks not only over a large dynamic range but also multiple samples in a reproducible manner will lead to a better understanding of the biological protein network and be a better clinical predictor than single protein measurements alone. Targeted proteomics is best suited to meet these needs. Multiple studies clearly demonstrated the reproducibility of SRM‐MS across laboratories 29, 30. SRM‐MS has been applied to quantify protein levels of liver tissue across 40 strains of BDX mouse 31 and quantitative trait analysis (QTL) of 78 *Saccharomyces cerevisiae* strains 32. Also, SRM assays are relatively easy to establish based either on prior knowledge (SRM assay repositories) or rapidly developed using whole protein digest 33. This is in contrast to establishing a new (batch of) affinity reagent.

## 2 Targeted MS considerations

### 2.1 Overview: targeted MS workflow

**Figure 3:**
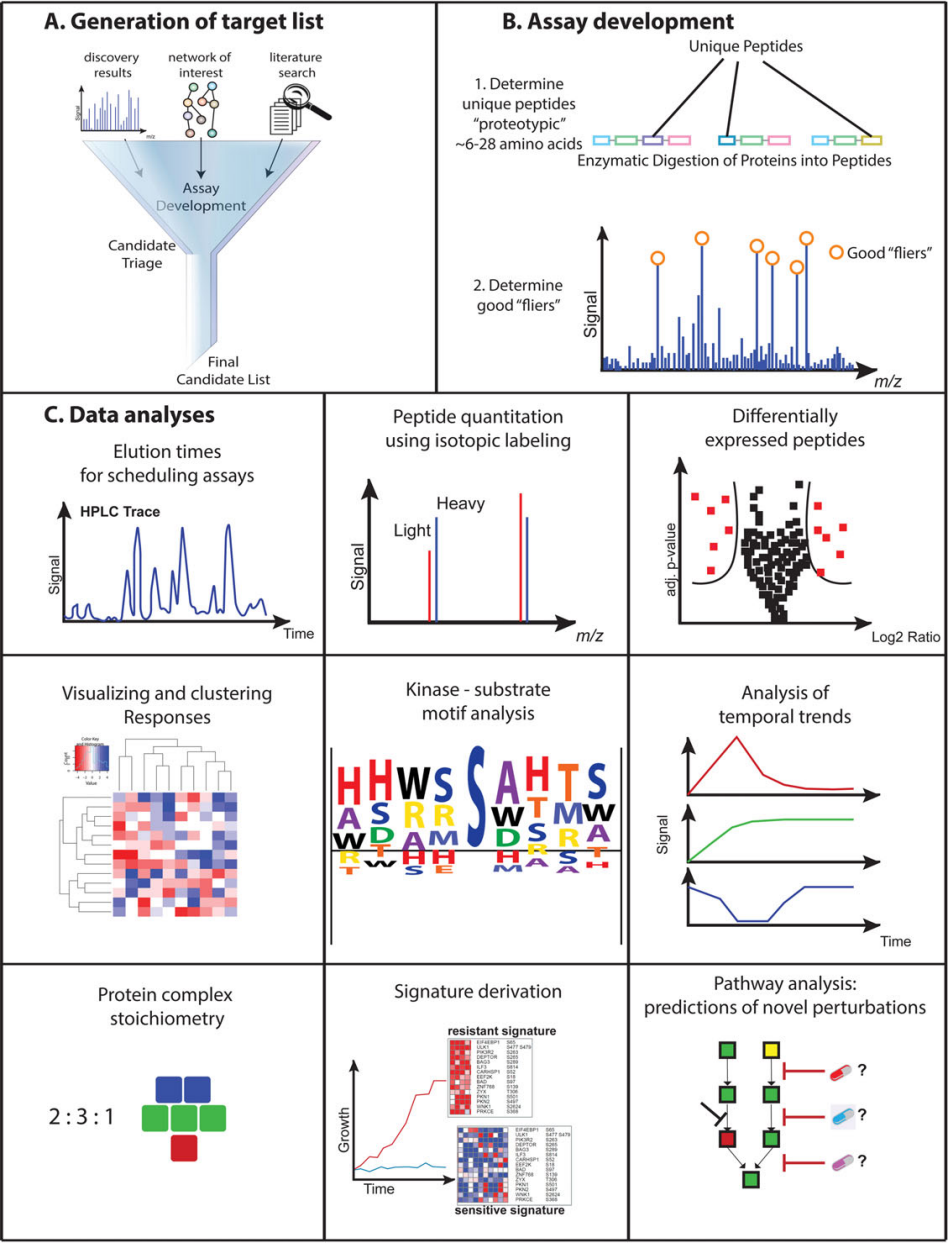
Typical targeted proteomics workflow. A. Discovery results from LC‐MS/MS experiments, protein network modeling and literature search typically form the basis to generate the final candidate list to be quantified by SRM. B. SRM assays for peptides are generated from extensive LC‐MS/MS experiments under consideration of proteotypic peptides generated and best performing transitions per peptide. C. Data anlysis starts with the primary LC‐MS/MS performance examination. If spiked in, stable isotope labeled peptides serve as reference for consistent quantification. Statistical analysis of peptides quantified serve to identify peptides, and therefore proteins, changing in abundance. Further analysis include the clustering of data corresponding to proteins quantified and condition. If multiple kinase substrates were quantified, a consensus motif analysis could identify novel substrate motifs of a kinase. In case the conditions are time course data, the abundance of proteins can be plotted as a function of time. Using SRM‐MS, protein stoichiometry of purified protein complexes can be determined (to be precise, this method requires newly synthesized externally calibrated reference peptides). The quantification of proteins and together with sample knowledge integration might lead to signatures which protein signature results in resistant or sensitive samples. The ultimate analysis is the protein network analysis leading to the prediction of novel perturbations.

A targeted MS‐based proteomics experiment consists of multiple steps and is schematically illustrated in Fig. 3. Specifically, the steps are (1) generation of a hypothesis, a target list of proteins to test the hypothesis and a fit‐for‐purpose quantitation strategy; (2) study design and experimental planning; (3) sample preparation; (4) method refinement; (5) data acquisition; (6) analysis and modeling 34, 35, 36. Bioinformatics and computational proteomics are part of each step of the workflow. Considerations regarding specificity, precision, and quantitative accuracy effect all steps of the targeted workflow and are discussed in more detail below.

### 2.2 Step 1: hypothesis development: selection of targets and quantitation strategy

The target list can be composed of several hundred peptides to be measured in a multiplexed fashion, allowing for a wide and versatile set of hypotheses to be tested. Typically the target list will be chosen from biomarker candidates found in a discovery profiling experiment, previous interest in a pathway, or from computational analysis identifying pathways and networks 8. The total number of analytes quantified per sample injection is typically 50–100 peptides.

In general, to test a hypothesis in a basic science model system, a larger set of proteins is typically measured, while quantifying biomarkers in a clinical setting for making treatment decisions usually involves a smaller set of proteins. The selection of a peptide quantification strategy should follow a fit‐for‐purpose approach to achieve the right level of specificity, precision, and quantitative accuracy as described recently in a three‐tiered system in Carr and colleagues 37. This system provides clear guidance with respect to the extent of analytical validation required for each major application type, termed Tier 1 to 3 in the publication 37. In this system, a “labeled internal standard” refers to the use of a consistent spike‐in for relative quantitation and most commonly consists of heavy‐labeled peptides, while a “reference standard” is as close as possible to the native protein in the sample and subject to all the same sample preparation steps, its absolute abundance is known, and calibration curves of dilutions can be established. The most demanding category are assays for clinical analysis (Tier 1) and require both labeled internal standards and reference standards, and may need to comply with additional regulatory requirements in each country. Common Tier 2 designs include measuring relative changes in protein expression levels and modifications after drug perturbation or disease for non‐clinical purposes. Exploratory studies (Tier 3) require some analytical validation, but do not require labeled internal standards or reference standards, and consequently have the lowest assay time development costs. Tier 3 type applications can proceed label‐free, at the cost of somewhat reduced quantitative accuracy. Analytical validation or reference standards are typically either chemically synthesized peptides or complex peptide mixtures containing stable isotopes 38.

All other steps of the SRM‐MS method development have been described in detail elsewhere 36, 39. Figure 3 depicts several of these steps and typical downstream analyses. It should be mentioned, that targeted proteomics described here is based on protease digest of whole proteins. The resulting peptides may be proteotypic and uniquely identify a single protein or protein isoform whereas other peptides may be derived from different proteins. In practice, some proteins may only be detected with a single peptide that is shared between closely related proteins, e.g. peptide LVVVGAGGVGK is shared between RASK, RASN, and RASH_HUMAN – rendering the quantification of a specific protein unreliable. A special case of proteotypic peptides are quantotypic peptides which are stoichiometric with total protein abundance and are not influenced by PTMs under the conditions tested 40. The choice of the quantified peptides and their suitability to serve as surrogates for protein identification and quantification is therefore an important aspect of targeting proteomics measurements.

## 3 Biology applications

### 3.1 Protein abundance studies

One of the earliest studies using targeted proteomics absolutely quantified G‐coupled receptor rhodopsin using chemically synthesized peptides as calibration standard to quantify endogenous levels of membrane bound rhodopsin 41. One of the earliest perturbed protein network study was carried out by Picotti and colleagues quantifying proteins of the Krebs Cycle under diauxic shift in *Saccharomyces cerevisiae* 16. The methodology quickly spread to medium sized target lists and has been used in a variety of basic biology applications, including pharmacology and developmental biology. Zhang and coworkers examined the abundance of two glutathione S‐transferase isoforms in human liver cytosol during detoxification 42. Heikkinen et al. quantified levels of P450, a drug modifying enzyme, in the drug model organism Beagle dog 43. In developmental biology, Betke et al. examined the differential localization and abundance of G‐beta and G‐gamma isoforms of G proteins in pre‐ and post‐synaptic fractions isolated from the cortex, cerebellum, hippocampus, and striatum of adult C57Bl6/J mice which showed significant differences in subcellular localization of different isoforms and provided an advance in understanding the roles of various subunits in different brain tissues 44. Pharmacological and toxicological examinations of 27 cytochrome P450 proteins in Balb/c mouse liver microsomes and tissue lysates from kidney, lung, intestine, heart, and brain across different developmental stages, including pregnancy were performed by Hersmann and colleagues 45.

Following the successful quantification of single proteins or small protein lists, assays for targeted proteomics were established for entire model organisms 16, 46, 47. Applications using medium to large‐sized target lists as is the case for the study of biological networks have pushed technology development. Chen and coworkers continued the exploratory studies of human liver by targeting 185 proteins previously detected in the Chinese Human Liver Proteome Project and confirmed the presence of 57 targets, 7 of which contained no information in PeptideAtlas, demonstrating the power of community efforts contributing to the completion of the Human Proteome Project 48. Worboys and coworkers recently developed assays targeting the human kinome, determining both proteotypic and quantotypic peptides for 21% of proteins in the human kinome 40. Targeted MS has also contributed to an understanding of development in zebrafish. Groh and coworkers used a combined global proteomics and computational approach to generate a candidate list of sex‐related development proteins and established roles for ILF2, ILF3, ZGC:195027 and other proteins 49.

Systems investigations of the response to perturbations by targeted proteomics are providing numerous insights in many higher eukaryotes. Zulak and coworkers investigated the effects of methyl jasmonate on terpene synthase enzyme induction and activity in protein extracts of Norway spruce, demonstrating a coordinated network of chemical defense response and a prime example of the robustness of biological systems 50. Choi and coworkers quantified eight adipokine proteins in response to hydrogen peroxide‐induced oxidative stress in adipocytes 51. Bisson and coworkers used affinity purification and affinity purification MS (AP‐MS) to quantify signaling dynamics of 90 proteins in the GRB2 interactome in HEK293T cells after growth‐factor stimulation 52. Xiang and coworkers used cell line models of multiple myeloma to investigate drug resistance of melphalan by comparing signaling, apoptosis‐regulating, and DNA repair component proteins, finding a nuclear factor‐kappaB signature 53.

Targeted proteomics is increasingly being used to quantify large numbers of proteins and addressing fundamental questions in biological networks. Sabido and coworkers measured 144 proteins in C57BL/6J and 129Sv mice subjected to various periods of high fat diet, revealing activation of either the peroxisomal beta‐oxidation pathway or the lipogenesis pathway in each strain, respectively 54. Kiel and coworkers quantified and localized 75% of an Erbb network of 198 signaling proteins across HEK293, MCF‐7, and keratinocytes, determining key quantitative parameters for cell‐type‐specific computational modeling in this fundamentally important network in cancer biology 8. The resulting protein signaling network is a complex, yet typical, network of signal transduction governed by protein abundance and protein‐protein interactions to convey phosphorylation signaling as seen in Fig. 1.

Targeted MS is increasingly being used to determine the stoichiometries of protein complexes a topic that is of similar importance to the understanding of biological systems through quantitative modeling. For example, monitoring the functional assembly of the human spliceosomal hPrp19/CDC5L complex under various conditions 55, the F_1_F_0_‐ATP synthase super‐assembly in H9c2 cardiomyoblasts undergoing cardiac‐like differentiation 56 or the determination of context‐dependent stoichiometry of the nuclear pore complex in various human cell lines, which showed unanticipated variability 57, 58.

Absolute quantification of proteins using targeted proteomics has considerably matured from single membrane protein quantification to functional stoichiometry determinations of protein complexes and quantification of protein networks. A pioneering approach was recently presented by Soste and coworkers: through literature search and computational prediction methods sentinel proteins were identified which report on the state of signaling pathways in a single SRM‐MS analysis. For *Saccharomyces cerevisiae* 157 proteins and 152 phosphorylated peptides were identified to reflect the status of the cellular signaling activity in a single analysis step, thus providing a broad overview of the state of numerous functional networks of the cell 59.

### 3.2 Post‐translational modification studies

Targeted MS has proven invaluable for the study of PTMs. Glinksi and coworkers examined multisite phosphorylation of trehalose‐6‐phosphate synthase isozymes in vitro in Arabidopsis 60. The importance of multisite protein phosphorylation and the value of quantifying it by targeted MS were recently shown for the connexin family of proteins and are described in a review by Chen and coworkers 61. Danielson and coworkers used antibodies specific for 3‐nitrotyrosine to quantify the levels of this modification in alpha‐synuclein residues 62. Held and coworkers developed a new method for studying oxidation in response to reactive oxygen species, termed oxMRM 63. In a tour‐de‐force application of their method, they examined site‐specific cysteine oxidation status of endogenous p53, finding that residue C182 at the dimerization interface of the DNA‐binding domain is susceptible to diamide oxidation. Huang and coworkers studied the effects of K63 polyubiquitination of EGFR on its endocytosis and post‐endocytotic sorting as mediated by ubiquitin adaptors 64. Darwanto and coworkers quantified H2B ubiquitination and H3 K79 methylation in the U937 human leukemia cell line and proposed a crosstalk regulatory mechanism between these two modifications 65. Wolf‐Yadlin and coworkers examined an EFGR network of 222 tyrosine phosphopeptides across seven time points following EGF stimulation of 184A1 HMEC cells, demonstrating excellent sensitivity, robust quantitation, and throughput 66. A useful case study and tutorial for targeted proteomics with enrichment is presented in Rardin and coworkers, in which they detail a method for measuring lysine acetylated peptides from mitochondria in mouse liver and targeted quantitation of a lysine acetylation site in succinate dehydrogenase A 67.

## 4 Clinical applications

### 4.1 Introduction to clinical applications

Clinical applications aim to translate new discoveries and technologies into improving patient outcomes 68 or to increase the understanding of biochemical processes underlying disease etiology. For several decades MS has played a key role in clinical chemistry, particularly for quantifying metabolites and hormones 69, 70. More recently, much work has gone into the development of targeted proteomics methods supporting clinical studies. Specific issues addressed include handling complexities of tissues and bodily fluids; agreement of community standards; formation of multi‐disciplinary research teams, and consortia, such as the Clinical Proteomics Tumor Analysis Consortium (CPTAC); demonstration of inter‐ and intra‐laboratory reproducibility; and proof‐of‐concept studies in a variety of clinical applications showing the feasibility of generating new clinical tests from biomarker discovery.

### 4.2 Biomarker development pipeline

The full pipeline of translating biomarker discovery studies into clinical tests with demonstrated health benefits is a multi‐year process that is costly, uncertain, and arduous and, because researchers and projects can easily get stuck in the middle of biomarker development projects, has been likened to a tar pit 71, 72. Moreover, there are a limited number of successful biomarkers approved across all technology platforms, and considerable controversy even surrounds the success stories, such as PSA testing 73. Although not the focus of this review, it is crucial to note that applied statistical testing methodology for biomarker development has itself advanced alongside clinical chemistry 73. A conceptual guide to overcoming biomarker challenges is presented by Rifai and coworkers in detail. They define: (i) candidate discovery; (ii) qualification; (iii) verification; (iv) research assay optimization; (v) biomarker validation; (vi) commercialization 72. As mentioned above, peptide quantification strategy should follow a fit‐for‐purpose approach to achieve the right level of specificity, precision, and quantitative accuracy as described in a three‐tiered system in Carr and colleagues 37. Further, findings of promising biomarkers or signatures from preclinical studies should be followed by clinical testing. The bulk of recent work has focused on candidate discovery and overcoming the associated challenges related to tissues and bodily fluids. Targeted proteomics can be used to validate biomarkers found in a project's discovery phase across many patient samples with high accuracy and reproducibility 29, 74. Cima and coworkers successfully applied this strategy by initially using shotgun data to establish a protein list of 44 candidates. Consistent quantification of these 44 proteins across a large patient cohort allowed the establishment of four N‐glycosylated protein makers which differentiate patients with a Gleasson score above or below 7 from blood serum 75.

### 4.3 Community efforts and the clinical proteomics technology assessment for cancer

The complexity in tissues and body fluids presents an enormous signal‐to‐noise ratio problem 34. The Human Plasma Proteome Peptide Atlas was launched to provide a knowledge‐base for targeted assays 15, 76. Inspiration for the use of these assays in the clinical setting comes from the small molecule clinical chemistry community where targeted MS is the gold standard for clinical assays quantifying inborn errors of metabolism, drugs and their metabolites, and steroids and biogenic amines 69. In 2006 the National Cancer Institute (USA) started the Clinical Proteomic Technology Assessment for Cancer (CPTAC) with the aims of evaluating targeted and discovery technologies for quantitative analysis in tissues and biofluids. This program was renewed in 2011 as the CPTAC, which began focusing on applications 77. CPTAC member laboratories applied standardized methods of multiple reaction monitoring (MRM) and demonstrated reproducibility, precision, sensitive quantitation in tissues and biofluids 78. Cox and coworkers performed a similar interlaboratory precision study of IGF‐1 in plasma across 130 healthy human samples and 22 samples from patients with acromegaly, finding excellent reproducibility 79. In summary, targeted proteomics has already established its cross‐laboratory reproducibility and robustness and will play a major role in validating protein biomarker across large patient cohorts.

### 4.4 Technology development and enrichment strategies

Over the last decade advances in targeted proteomics have lead to assay sensitivity for plasma proteins in the low ng/ml range. In 2004, Kuhn and coworkers used a depletion of abundant proteins strategy and size exclusion fractionation to quantitate C‐reactive protein, a diagnostic marker for rheumatoid arthritis 80. Anderson and Hunter targeted 53 medium and high abundant proteins in plasma, leading to assays for 47 of the proteins covering 4.5 orders of magnitude with minimal sample preparation 81. Keshishian and coworkers developed multiplexed assays for six plasma proteins present in 1–10 ng/ml using strong cation exchange chromatography and major abundant protein depletion, but without immunoaffininty enrichment, demonstrating that the abundance range of typical candidate biomarkers (ng/ml) is achievable with targeted proteomics 82. Fortin and coworkers also achieved ng/ml sensitivity without immunoaffinity enrichment by quantitating prostate‐specific antigen (PSA) in sera from patients with prostate cancer or benign hyperplasia; moreover, their MS results agreed with established ELISA tests for PSA 83. Shi and coworkers used an antibody‐free approach termed PRISM (high‐pressure, high‐resolution separations coupled with intelligent selection and multiplexing) in order to quantitate PSA levels in the range of 50–100 pg/ml, also with excellent correlation to clinical immunoassays 84. Shi and coworkers applied the PRISM targeted proteomics to quantitate AGR2 in human urine at serum at concentrations of approximately 130 pg/ml and 10 pg per 100 ug of total protein mass in urine, respectively, and found in a proof‐of‐concept study of 37 urine samples that AGR2/PSA concentration ratios can distinguish noncancer and cancer 85. Fallon and coworkers developed assays for 14 UGT1As and UGT2Bs across 60 human liver microsomes and matching S9 samples to evaluate metabolism in drug development 86.

Technology development has also advanced in labeling incorporation technology and throughput. Zhao and coworkers developed a synthetic peptide strategy using ^18^O labeling strategy for SID‐MRM‐MS with the ability to produce synthetic peptides for use as internal quantitation standards in only 1 h with excellent stability 87. They then utilized these labeled peptides for absolute quantitation of candidate hepatocellular carcinoma biomarkers vitronectin and clusterin in undepleted serum samples. Martinez‐Morillo and coworkers developed assays for absolute quantification of apolipoprotein E isoforms in cerebrospinal fluid and plasma, which are important in lipid metabolism in the central nervous system and are associated with coronary atherosclerosis and Alzheimer's disease, and also assessed the effects of chemical modifications on selected target peptides’ quantitation 88. Tang and coworkers used major protein depletion and 1D gel separation, starting with less than 100 μl of serum, obtaining reproducible quantitation without internal standards down to 200 pg/ml in assays for PRDX6, ADAM12, PAEP, CGB, and CTSD, which demonstrates that their GeLC‐MRM workflow has sufficient throughput, sensitivity, and costs for an initial screening of large numbers of candidate biomarkers 89.

Technology development has gone into using PTMs as handle to enrich for partially low abundant proteins. One such enrichment strategy is the enrichment of N‐glycosylated peptides and the subsequent PNGase F‐catalyzed conversion of Asn to Asp using solid state extraction method 90, 91 a method which was successfully applied to serum samples 75, 92. Upon purification, only the modified peptide is quantified. Stahl‐Zeng and coworkers applied minimal fractionation of isolated N‐glycosites to quantitate plasma proteins over five orders of magnitude, reaching sub‐ng/ml range 93. Zawadzka and coworkers used both targeted and discovery proteomics to quantify a set of approximately 60 phosphopeptides from healthy human plasma following offline chromatography and immobilized metal ion affinity chromatography for phosphopeptide enrichment 94. Further advances in targeted proteomics of N‐glycosites will undoubtedly bring the N‐glyco Atlas providing SRM assays for 5568 N‐to‐D‐modified peptide sequences 95. These SRM‐assays were applied to prostate cancer tissue samples to determine aggressiveness of tumors using targeted extraction of peptide sequences from SWATH‐MS maps 96.

Another strategy is the enrichment microparticles 97, 98 or exosomes (or extracellular vesicles) articles from bodily fluids, e.g. human urin or serum, followed by protein isolation and quantification of peptides using SRM. The exosome enrichment strategy was applied to various diseases ranging from bladder cancer 99 over diabetic nephropathy 100 to detecting *Mycobacterium tuberculosis* peptides in serum of patients with active or latent *M. tuberculosis* infection 101.

### 4.5 Immuno‐affinity SRM

While immuno‐reagents are commonly used to deplete the most abundant proteins in plasma they can also be used for enrichment of low abundant proteins. A commonly used technique is known as stable isotope standards and capture by anti‐peptide antibodies (SISCAPA) 102. Dupuis and coworkers quantified staphylococcal enterotoxins in foods by applying a combination immunocapture and protein standard absolute quantification (PSAQ) method, which uses isotope‐labeled enterotoxins as internal standards 103. Oe and coworkers used immune capture of amyloid betas in cerebral spinal fluid as potential biomarkers of Alzheimer's disease achieving limits of quantitation down to 200 pg/ml 104. Berna and coworkers used immune capture to develop assays to quantify myosin light chain 1 in rat serum as a biomarker of cardiac necrosis to predict drug‐induced cardiotoxicity over a range of 0.13 – 6.62 nM 105. Nicol and coworkers used an immunoaffinity approach to quantify carincoembryonic antigen (CEA), secretory leukocyte peptidase inhibitor, tissue factor pathway inhibitor 1,2 (TFPI/TFPI2), and metalloproteinase inhibitor 1 (TIMP1) in sera samples from lung cancer patients down to low ng/ml levels 106. Hoofnagle and coworkers quantified the cancer marker thyroglobulin in serum using an immunoaffinity approach down to a limit of detection of 2.6 ng/ml 107.

Immunoaffinity targeted proteomics can also be used in combination with additional enrichment techniques to reach low limits of quantitation. Ahn and coworkers used a variation of SISCAPA with a combination of phytohemagglutinin‐L_4_ (L‐PHA) for N‐linked glycan capture and a monoclonal anti‐peptide TIMP1 antibody conjugated to magnetic beads to quantitate the cancer candidate biomarker TIMP1, which is present at approximately 0.8 ng/ml in serum using only 1.7 μl of serum from a patient with colorectal cancer 108. Using online chromatography for affinity capture instead of offline magnetic beads, Neubert and coworkers quantified a marker for gastroesophageal reflux disease, pepsin/pepsinogen, in the saliva of healthy volunteers down to a range of 0.17 to 0.67 ng/ml, providing the most sensitive and specific test to date 109. A few years later, Neubert and coworkers made use of robotic sample preparation and sequential immunoaffinity capture of the protein NGF using magnetic beads followed by online affinity capture of a signature NGF peptide, which is the first report of this combination technique, and allowed for quantitation of NGF to 7.03 to 450 pg/ml in a clinical trial for chronic pain 110.

Immunoaffinity techniques can also be useful for quantitating proteins in the presence of autoantibodies. Thyroglobulin (Tg) is used to monitor patients after treatment for differentiated thyroid carcinoma and is often accompanied by the presence of auto‐antibodies that interfere with immunoassays. Kushnir and coworkers overcame these challenges by enriching serum samples with a rabbit polyclonal antibody for Tg and immunoaffinity purification of a signature Tg peptide, with a lower limit of quantitation of 0.5 ng/ml 111.

Immunoaffinity techniques are also amenable to multiplexing. Using an approach known as mass spectrometric immunoassay (MSIA), Krastins and coworkers rapidly developed assays for 16 different target proteins and their isoforms across seven different clinically important areas and ranging in concentration from pg/ml to ng/ml in bona fide clinical samples 112. Recently, Peterman and coworkers applied the MSIA approach to detect insulin and its analogues using a pan‐insulin antibody over a range from 1.5 to 960 pM 113. Other immune‐MRM efforts are conducted by the Paulovich laboratory to overcome limit of detection issues in complex samples and eliminate the current bottleneck of translating biomarkers found in basic science studies to clinical practice 114. Further advances in the field concern the generation of immuno‐MRM monoclonal antibodies suitable for SRM and conventional antibody applications 115.

### 4.6 Cardiovascular disease applications

Perhaps the main advantage of targeted MS‐based proteomics over ELISA assays is their multiplexing capability, which is of key importance for biomarker development in disease applications with many putative biomarkers such as cardiovascular disease and cancer. Kuzyk and coworkers developed a mixture of 45 peptide standards in EDTA‐plasma without affinity depletion or enrichment and found that 31 of the 45 are putative markers of cardiovascular disease 116. Keshishian and coworkers developed quantitative assays without immunoaffinity enrichment for six proteins of clinical relevance to cardiac injury ranging from 2 to 15 ng/ml and measured these proteins across three time points in six patients undergoing alcohol septal ablation for hypertrophic obstructive cardiomyopathy 117. Addona and coworkers used a combination of discovery and targeted proteomics in the context of planned myocardial infarction (PMI) for treatment of hypertrophic cardiomyopathy and myocardial infarction (MI) 118. Samples of blood directly from patient hearts before, during, and after PMI allowed identification of 121 candidate biomarker proteins, over 100 of which were novel. Targeted proteomics was then applied to peripheral plasma from controls and patients with PMI or MI, suggesting verification of candidate biomarkers 118. Huillet and coworkers used the PSAQ‐SRM approach to quantitate cardiovascular disease biomarkers LDH‐B, CKMB, myoglobin, and troponin I, in serum samples from myocardial infarction patients 119. Domanski and coworkers developed assays with 135 stable‐isotope labeled peptides for quantitation of 67 candidate biomarkers of cardiovascular disease spanning the top seven most abundant orders of magnitude of concentration in whole plasma with a 30 min assay time, performing 85 technical replicates, which showed excellent sensitivity and retention time accuracy 120. Further, solid state N‐glyco enrichment strategies can be used to enrich to monitor cardiac resynchronization therapy in serum from a canine model 121.

### 4.7 Cancer applications

The heterogeneity and complexity present in the numerous types of cancer presents an enormous opportunity but significant challenges for targeted proteomics to make transformative contributions. Much technology development has gone into tissue preparation and quantitation of candidate biomarkers addressing the dual challenges of using a limited amount of material in which the candidate biomarker may also be low abundant. DeSouza and coworkers quantified a marker of endometrial cancer, pyruvate kinase M1/M2, in biopsied tissue at 85 nmol/g compared to 21–26 nmol/g in nonmalignant tissue using the mTRAQ labeling method, compared to only a 2x elevation initially determined by discovery iTRAQ proteomics, suggesting that the dynamic range for quantitation may be compressed in discovery scans 122. Chen and coworkers developed quantitative assays for 22 proteins in the Wnt/beta‐catenin signaling pathway in colon cancer cell lines and applied them to frozen colon tissue sections and laser capture microdissected tumor cells 123. Elschenbroich and coworkers combined discovery and targeted proteomics to develop assays for serous‐type epithelial ovarian cancer discovering a panel of 51 candidate proteins and then using synthetic peptides (13 proteins) and stable isotope label standards (four proteins) for targeted quantification in ascites in serum, providing proof‐of‐concept validation for this strategy 124. Remily‐Wood and coworkers used pathway analysis to develop 95 quantitative assays including synthetic peptide standards for proteins of interest in colon, lung, melanoma, leukemias, and myelomas, which are published online in a Quantitative Assay Database 125. Selevsek and coworkers developed multiplexed assays with stable isotope dilution standards to analyze 16 proteins associated with bladder cancer in urine with excellent analytical performance and limits of quantitation limits in the low ng/ml range 126.

Clinical proteomics in cancer has also addressed challenges of integrating genomic data such as mutations. Wang and coworkers demonstrated that altered protein products resulting from somatic mutations can be quantified by targeted proteomics by developing assays for Ras proteins and applying them in colorectal and pancreatic tumor tissue and premalignant pancreatic cyst fluids 127. He and coworkers recently developed assays to quantitate ERG isoforms in TMPRSS2‐ERG positive VCaP cell line and two prostate cancer tissue samples 128.

In 2011 a complete verification pipeline of biomarker candidates in plasma was presented in a tour‐de‐force study by Whiteaker and coworkers 129. They integrated 13 datasets from discovery proteomics and genomics to arrive at >1000 candidate proteins in a mouse model of breast cancer and used data‐dependent prioritization to triage candidates, developing assays for 88 proteins evaluated across 80 plasma samples, finding 36 over‐expressed proteins with excellent analytical performance. Their data‐dependent triage of candidates used a MS approach termed accurate inclusion mass screening (AIMS), which is essentially an efficient bridge from discovery to targeted proteomics that uses inclusion‐list dependent acquisition on an orbitrap mass spectrometer to verify the presence of a candidate (Jaffe et al., 2008).

Hüttenhain and coworkers also completed a tour‐de‐force complete verification pipeline of biomarker candidates by generating assays for 1000 cancer‐related proteins and used a data‐dependent triage strategy by first examining candidate proteins’ detectability in plasma and urine samples 130. They subsequently detected 182 proteins in depleted plasma, spanning five orders of magnitude in abundance and 408 proteins in urine. They then profiled 83 patient plasma samples for 34 of the candidate biomarkers using heavy‐labeled synthetic peptides, finding that their targeted proteomics assays allowed for reproducible quantitation.

Biomarker discovery and verification has also been pursued for post‐translationally modified proteins. Cima and coworkers followed a two‐stage strategy for biomarker discovery, starting with a discovery scan comparing the serum N‐linked glycoproteome of PTEN conditional knockout model of prostate cancer to wild‐type and then developed targeted assays for 39 human orthologs, which were then quantified in the sera of 143 prostate cancer patients and controls over an abundance range of six orders of magnitude 75. Computational analysis derived a signature for diagnosis and prognosis of prostate cancer. In a followup study, Kalin and coworkers used the N‐linked glycoprotein capture and assays to quantitate candidate biomarkers in sera of 57 patients with metastatic prostate cancer 131. Computational analysis integrated known prognostic factors with candidate N‐linked glycoproteins derived new nomograms with potentially improved accuracy. Cerciello and coworkers used a three stage approach for identification of biomarker candidates in the serum of malignant pleural mesothelioma 132. First they screened a collection of relevant cell lines and discovered 125 candidate cell surface N‐linked glycoprotein peptides. They then developed assays for 51 candidates and screened sera from five patients. In the third stage, the diagnostic potential of 51 candidate peptides was assessed through targeted proteomics of 75 patient sera samples, with a balanced design of 25 malignant pleural mesothelioma, 25 healthy donors, and 25 non‐small cell lung cancer patients. Computational analysis found a seven glycopeptide signature for malignant pleural mesothelioma with better discrimination than the FDA approved ELISA assay for mesothelin (Mesomark®).

Targeted proteomics has been applied successfully to formalin‐fixed, paraffin‐embedded (SFPE) tissue. Sprung and coworkers quantified 114 peptides in FFPE clear cell renal cell carcinomas and Her2 overexpression in FFPE breast cancer samples and determined by comparison with cell lines that lysine‐containing peptides can be used for quantitation and the feasibility of performing targeted proteomics studies on FFPE tissues 133. Takadate and coworkers performed a discovery scan on eight FFPE resectable, node‐positive pancreatic ductal adenocarcinoma, and five FFPE noncancerous pancreatic ducts and selected 170 of 1229 candidate proteins for targeted assay development, which they applied to a cohort of 87 cases, finding 14 overexpressed proteins in the poor vs. better outcome groups, ultimately nominating four proteins as prognostic markers: ECH1, OLFM4, STML2, and GTR1 134. Pan and coworkers developed assays for five candidate biomarkers of pancreatic cancer and quantified them in plasma obtained from 20 healthy patients, 20 patients with chronic pancreatitis, 20 with early stage pancreatic ductal adenocarcinoma, finding that three of the markers gelsolin, lumican, and tissue inhibitor of metalloproteinase 1 can distinguish pancreatic cancer from controls 135.

## 5 Conclusion

Targeted proteomics is now a well‐established tool for quantitative proteomics and is most useful when researchers can identify a medium‐to‐large target list (for examples, see Table 1). Through selection of sentinel proteins, the activation state of a given cellular process can be monitored 59, thus extending the coverage of SRM. To date, the technology has been mainly applied to clinical proteomics and less often to basic biological network questions. In the future, targeted proteomics might play a more vital role in characterizing critical post‐translationally modified amino acid residues and larger biological networks in a large diversity of developmental stages, disease, contexts, and perturbations. Targeted proteomics should continue to advance in the clinical setting. Targeted proteomics can contribute to an understanding of networks’ responses to treatment, such as examination of phosphoprotein response in tumor biopsies before and after treatment. With respect to biomarkers, large collaborative projects such as The Cancer Genome Atlas (TCGA) and CPTAC are finding numerous biomarker candidates, which should be pursued through a sustained commitment to completing the entire biomarker pipeline, which should either eliminate candidates or result in validated and valuable biomarkers translated to clinical practice.

**Table 1:** Key studies in targeted MS Some key studies chosen from the reference list covering a wide range of applications of SRM‐MS

Study	Description	Assays successfully developed
*Biological applications*		
Hersmann 2014	Quantitation of cytochrome P450's across developmental stages and tissues	27 cytochrome P450 proteins
Chen 2014	Human liver proteome	57 out of 185 human liver proteins
Worboys 2014	Human kinome	790 proteotypic peptides targeting 196 human kinases – 80% with good quantotypic properties
Wolf‐Yadlin 2007	EFGR network across seven time points following EGF stimulation of 184A1 HMEC cells	222 tyrosine phosphopeptides in EGFR network
Sabido 2013	Networks activated by a high fat different across mice strains	144 metabolism related proteins
Bisetto 2013	F_1_F_0_‐ATP synthase super‐assembly in H9c2 cardiomyoblasts undergoing differentiation	Complex stoichiometry determined
Kiel 2014	Erbb network in human cancer cell lines	75% of 198 proteins in the network
*Clinical applications*		
Huttenhain 2013	Glycosites	5568 N‐glycosites
Krastins 2013	Samples from seven different clinical areas	16 target proteins spanning pg/ml to ng/ml
Addona 2011	Planned myocardial infarction	121 biomarker candidates
Domanski 2012	Cardiovascular disease	67 candidate biomarkers
He 2014	ERG isoforms in prostate tissue	Multiple ERG isoforms
Whiteaker 2011	Biomarker discovery using a mouse model of breast cancer	88 proteins in 80 plasma samples; 57‐plex SRM and 31‐plex immuno‐SRM
Huttenhain 2012	Cancer‐related proteins in plasma and urine	182 proteins in depleted plasma; 408 in urine

Advances in MS instrumentation will undoubtedly impact the field towards consistently quantifying more proteins per sample. Similar to SRM‐MS, PRM‐MS (parallel reaction monitoring mass spectrometry) takes advantage of a mass spectrometer which first selects precursors using a triple quadrupole and subsequently fragment ions are mass analyzed in an orbitrap 136. Using the same instrumentation as PRM‐MS, the instrument method can be adapted to allow for data independent acquisition 137, 138. Instead of coupling a quadrupole with an orbitrap mass analyzer, SWATH‐MS (sequential window acquisition of all theoretical masses) was implemented on a mass spectrometer which selects the precursors with a quadrupole and all fragment ions are analyzed by TOF 139. First clinical studies using SWATH‐MS demonstrated the usefulness of the method: Liu and colleagues concluded from the analysis of serum proteins of a longitudinal twin study that clinical serum biomarkers should be calibrated against genetic background and age adjusted 140.

*The authors have declared no conflict of interest*.
